# An Evaluation of the Appropriateness of Initial Enoxaparin Dosing Among Pediatric Patients

**DOI:** 10.7759/cureus.48830

**Published:** 2023-11-15

**Authors:** Abdullah Altuwayjiri, Amirah AlDarwish, Renad Alshuraim

**Affiliations:** 1 Pharmaceutical Services Department, King Saud Medical City, Riyadh, SAU; 2 Pharmaceutical Department, King Fahad Medical City, Riyadh, SAU

**Keywords:** enoxaparin, anti-factor xa, deep vein thrombosis, pediatric, neonate

## Abstract

Background

Enoxaparin is a low molecular weight heparin that irreversibly inactivates factor Xa leading to the inhibition of clot formation. Despite the non-FDA approval in pediatrics, enoxaparin is recommended with an initial dose of 1.5mg/kg/q12hrs for patients aged ≤ 2 months and 1mg/kg/q12hrs for patients > 2 months, targeting therapeutic anti-Xa with a range of 0.5 to 1 units/mL. Due to more rapid clearance in pediatrics, our study aims to assess the need for initial higher doses than recommended by the guideline to reach the target anti-Xa level.

Methods

A retrospective chart review of all pediatric patients under all specialties who were treated with enoxaparin either in inpatient or outpatient settings between February 2021 and June 2022 at children's specialized hospital and meet the inclusion criteria, including age ≤ 15 years old and treated with enoxaparin with initial dose according to the American College of Chest Physicians (CHEST) guideline, while patients who received prophylaxis doses did not have anti-Xa levels or creatinine clearance < 30 mL/min/1.73m^2^ were excluded. Demographic data, laboratory data, and enoxaparin dosing were all collected to assess whether the initial enoxaparin dose will result in a therapeutic level as a primary endpoint and secondary endpoints including the average enoxaparin dose required to achieve the therapeutic level and to report any side effects.

All data were entered and analyzed using the Statistical Package for the Social Sciences (IBM SPSS Statistics for Windows, IBM Corp., Version 25, Armonk, NY), and all categorical variables were reported as frequency and percentage while continuous variables expressed as mean ± SD and the study was approved by our research center institutional review board (IRB).

Results

Thirty patients were included in the study (17 males), 10 patients were aged ≤ 2 months, four were between 3 and 12 months and 16 were > 12 months, most of the patients received enoxaparin for deep vein thrombosis. In the majority of patients (76.7%), the initial dose failed to achieve the target anti-Xa while a mean dose of 2 mg/kg/q12hrs in patients ≤ 2 months, 1.7mg/kg/q12hrs in patients 3-12 months and 1.3 mg/kg/q12hrs in patients > 12 months was sufficient to reach the target level. After achieving a therapeutic anti-Xa level, only one patient experienced major bleeding while four patients experienced minor bleeding, no edema or thrombocytopenia were reported.

Conclusion

In conclusion, initiating enoxaparin according to the recommended dose by the guideline failed to achieve target anti-Xa in the majority of patients which necessitates starting enoxaparin with initial higher doses according to the patient's age to provide more prompt achievement of target anti-Xa.

## Introduction

Enoxaparin is a low molecular weight heparin (LMWH) and its use in pediatrics has increased significantly in the last years to treat thromboembolic events. Enoxaparin works by binding to and stimulating the activity of antithrombin III, which in turn will form a complex that irreversibly inactivates factor Xa leading to the inhibition of clot formation. As compared to unfractionated heparin (UH), enoxaparin poses many advantages, including ease of subcutaneous administration (SC) which allows outpatient treatment, lack of drug-drug interactions, more predictable pharmacokinetic parameters necessitating less need for frequent monitoring, longer half-life allowing less frequent administration, 100% bioavailability after SC administration, complete renal clearance and less protein binding, but more interpatient variability in the coagulation effect have been noticed with the weight-based dosing as compared to adult patients [1-3]. According to the American Society of Hematology and the American College of Chest Physicians (CHEST) guidelines, enoxaparin is recommended in the pediatric population to manage provoked and idiopathic deep vein thrombosis (DVT), ischemic stroke and patients with protein C deficiency with an initial dose of 1.5mg/kg/dose q12hrs for patients age ≤ 2 months and 1mg/kg/dose q12hrs for patient > 2 months, targeting therapeutic anti-Xa with range of 0.5 to 1.0 units/mL in sample taken 4 to 6 h or 0.5 to 0.8 units/mL in a sample taken 2 to 6 h after achieving the steady-state concentration that is typically reached after the third or fourth dose [2,4]. Despite the non-FDA approval, the safety of enoxaparin in pediatric patients was assessed in many studies, the major bleeding rate appears to be low in stable patients ranging from 0% to 5% and the minor bleeding rate was reported to be around 16% and there are no reported cases for heparin-induced thrombocytopenia, osteoporosis, or any other hypersensitivity reactions [2,5]. Enoxaparin use in kidney impairment has been evaluated in a pharmacokinetics (PK) study and found that approximately about 30% reduction in the initial dose is required as the clearance is decreased by 30% in patients with creatinine clearance (CrCl) < 30mL/min [6].

The pediatric population was found to have more rapid clearance of enoxaparin than adult patients, due to this our study aims to assess the need for initial higher doses than recommended by the guideline to reach the target therapeutic anti-Xa level.

## Materials and methods

Patient population

This single-center study was performed with the approval of the Institutional Review Board. We identified individuals under 15 years of age who had been hospitalized between February 2021 and March 2022 and had received enoxaparin treatment doses, using the hospital record system. Patients who did not have reported anti-factor Xa concentrations had a creatinine clearance of less than 30 ml/min/1.73 m^2^, or had received prophylactic doses of enoxaparin were excluded from the study.

Data collection

This study was a retrospective chart review. Demographic data, including the patient's age, gender, height, and weight, were collected. Laboratory data such as anti-Xa levels, complete blood count (CBC), alanine transaminase (ALT), and aspartate transaminase (AST) were recorded. The study also documented signs and symptoms of major bleeding, defined as a decrease in hemoglobin levels of 2 g/dL or more, and symptomatic bleeding in a major organ, also reporting of minor bleeding was documented. Information on the initial dose of enoxaparin, adjusted dose, and target dose for reaching therapeutic levels was collected. An anti-factor Xa level is considered therapeutic if within the range of 0.5 to 1 units/mL, drawn four to six hours after dose administration. If multiple doses resulted in a therapeutic anti-factor Xa level, the lowest dose was recorded.

Data analysis

A descriptive analysis was carried out in which categorical variables were expressed as absolute and relative frequencies, and continuous variables as means (SD). The X2 was used to compare proportions in independent groups. Binary logistic regression analyses were used to identify factors associated with enoxaparin and dose. Factors were selected with a backward stepwise method. Unstandardized regression coefficients (β) and odds ratios (ORs) and their 95% confidence intervals (CIs) were used to quantify the associations between variables.

## Results

Thirty patients were included in the study and the majority were male (56.6%), 10 patients were aged ≤ 2 months, four were between 3 and 12 months, and 16 were > 12 months, most of the patients received enoxaparin for DVT (Table 1).

**Table 1:** Patient demographics DVT: deep vein thrombosis, LV: left ventricular thrombosis

Variables	Description	N (n%)
Age Group (Months)	≤ 2	10 (30%)
	3-12	4 (16.7%)
	> 12	16 (53.3%)
Gender	Male	17 (56.7%)
	Female	13 (43.3%)
Indication	DVT	28 (93.3%)
	LV	1 (3.3%)
	Protein C deficiency	1 (3.3%)

In the majority of patients (76.7%), the initial dose failed to achieve the target anti-Xa and most of the patients were less than two months (Table 2).

**Table 2:** Rate of reaching target Xa with initial dose according to the age

Variables	Description	Initial dose and therapeutic Xa level	
		Therapeutic level	Non-therapeutic level
Age Group (Months)	< 2	1	8
	2-12	0	5
	> 12	6	10

A dose of 2mg/kg/q12hrs in patients ≤ 2 months, 1.7mg/kg/q12hrs in patients 3-12 months, and 1.3 mg/kg/q12hrs in patients > 12 months was sufficient to reach the target level (Table 3).

**Table 3:** Association between dose required to target Xa level and age group

Age	Mean dose achieved target Xa
≤ 2 months	2 mg/kg/q12hrs
3-12 months	1.7 mg/kg/q12hrs
> 12 months	1.3 mg/kg/q12hrs

After achieving a therapeutic anti-Xa level, only one patient experienced major bleeding while four patients experienced minor bleeding, no edema or thrombocytopenia were reported.

## Discussion

Unlike the use of enoxaparin in the adult population, there is a consensus on the unpredictability of the anticoagulant effect with weight adjustment, and recommended that due to considerable interpatient dose differences, children must be routinely monitoring anti-Xa level. However, there is still a controversy on the optimal initial dose of enoxaparin in the pediatric population. Results of our study showed that the majority of our patients (76.7%) who received an initial dose of enoxaparin as recommended by CHEST/AHA guidelines, failed to achieve the target anti-Xa, whereas only (23.3%) achieved the therapeutic target. A subgroup analysis showed that a higher percentage of patients reached therapeutic levels were >12 months. On the other hand, only one patient reached the target in the group aged 2-12 months and none of the patients <2 months reached the therapeutic target with initial dosing. Variations in results might be due to differences in patient total number in each group.

A mean dose of 2 mg/kg q12hrs was reached in patients ≤ 2 months, and 1.7 mg/kg q12hrs mean dose was needed in patients 3-12 months. Whereas the mean dose was titrated up to 1.3 mg/kg/q12hrs dose in patients > 12 months, previously mentioned doses were sufficient to reach a therapeutic concentration. Comparing CHEST guidelines recommendation with our study outcomes there was a need for a 25% increase in initial dose in patients <2 months, whereas a 70% to 30% increase is needed in patients aged 2-12 and >12 months respectively. Our results matched the supporting evidence of the need for higher initial dosing in infants than in children and adults. However, there was a discrepancy in the targeted dose needed; this is due to a small number of patients enrolled and the difference in age groups among studies. A retrospective study conducted by LS Fung et al. (2010) on 150 pediatric patients with ages ranging from 1 month to 18 years found that the dose was required to achieve a therapeutic anti-Xa level higher than the dose recommended by the guideline with an average dose of 1.8 mg/kg q12h in patients < one month, 1.64 mg/kg q12hrs for one month to one year, 1.45 mg/kg q12hrs in patients 1-6 years and 1.05 mg/kg q12hrs > 6 years of age [7]. Another retrospective study was conducted by EW McCormick et al. (2015) to evaluate the safety and efficacy of enoxaparin in pediatric patients, 32 patients included in the study with ages of < 2 months to 17 years, and more than 60% of patients failed to achieve target therapeutic anticoagulation with the initial recommended dosing and the median therapeutic doses were reported to be 1.5 mg/kg q12hrs in patients aged <2 months, 1.5 mg/kg q12 in 2 months to <1 year, and one in 3 to 17 years [8]. A single-center, retrospective study conducted by JK Hicks et al. (2012) included 33 infants with age less than two months to evaluate the initial dose of enoxaparin and the mean dose required to reach the therapeutic level, only six patients (19.2%) achieved therapeutic anti-Xa level with initial dosing, and a mean dose of 2 mg/kg q12hrs was required to achieve the therapeutic level, another finding was that patients who were born prematurely required a higher enoxaparin dose 2.2 mg/kg/q12hrs than did those born at full-term gestation [9]. Moreover, the safety outcome of enoxaparin dosing was evaluated in our population, and the findings of our study indicate that there was only one patient who had a major bleeding event, one patient with bruises, and three patients who suffered from epistaxis.

There are several limitations to consider in this study. Firstly, the retrospective design on previously recorded electronic data may introduce uncontrollable individualized factors. Additionally, the unequal and small number of patients in the different age groups prevented us from making a statistical comparison of the median therapeutic doses across the groups. Furthermore, we did not assess whether there was actual resolution of the thrombi, which is an additional limitation of this study. Considering these limitations, it is recommended that a future multicenter study be conducted to provide more comprehensive data on dosing, monitoring, and efficacy outcomes of enoxaparin in pediatrics. Such a study would be valuable due to the variable pharmacokinetics and dynamics that accompany growth and development differences in children. This research could potentially lead to a revision of the recommended initial dose of enoxaparin, as the current dose has been shown to be insufficient in achieving therapeutic anti-Xa levels. Furthermore, age stratification should lead the enoxaparin dosing in pediatric patients rather than a general dose for a vast age group.

## Conclusions

Initiating enoxaparin according to the recommended dose by the guideline failed to achieve the target anti-Xa in the majority of patients which necessitates starting enoxaparin with initial higher doses according to the patient's age to provide more prompt achievement of target anti-Xa.
